# Mixed Antimony(V) Complexes with Different Sugars to Modulate the Oral Bioavailability of Pentavalent Antimonial Drugs

**DOI:** 10.3390/molecules19055478

**Published:** 2014-04-28

**Authors:** Weverson A. Ferreira, Arshad Islam, Aretha Priscilla S. Andrade, Flaviana R. Fernandes, Frédéric Frézard, Cynthia Demicheli

**Affiliations:** 1 Department of Chemistry, Institute of Exact Sciences – Federal University of Minas Gerais (UFMG), Av. Antônio Carlos 6627, Belo Horizonte 31270-901, MG, Brazil; 2 Department of Physiology and Biophysics, Institute of Biological Sciences – Federal University of Minas Gerais (UFMG), Av. Antônio Carlos 6627, Belo Horizonte 31270-901, MG, Brazil

**Keywords:** pentavalent antimonial drugs, meglumine antimoniate, leishmaniasis, maltose, ribose, cyclodextrin, oral

## Abstract

Previous studies have shown that the association of the drug meglumine antimoniate (MA) with β-cyclodextrin can improve its bioavailability by the oral route. In this work, ribose and maltose were investigated for their ability to form mixed or association complexes with MA, release MA and modulate the serum levels of Sb after oral administration in mice. Analysis of the MA/ribose composition by high performance liquid chromatography coupled to mass spectrometry (LCMS-IT-TOF) revealed the presence of mixed meglumine-Sb-ribose and Sb-ribose complexes. Analysis of the MA/maltose composition suggested the formation of MA-maltose association compounds. Circular dichroism characterization of these compositions following dilution in water at 37 °C suggested a partial and slow dissociation of the association compounds. When the MA/ribose composition was administered orally and compared to MA, the serum concentration of Sb was significantly lower after 1 h and greater after 3 h. On the other hand, the MA/maltose composition showed similar serum Sb concentration after 1 h and higher level of Sb after 3 h, when compared to MA. In conclusion, the present study has demonstrated the formation of mixed or association complexes of MA with sugars, such as maltose and ribose, which promoted sustained serum level of Sb after oral administration.

## 1. Introduction

Leishmaniases are infective tropical parasitic diseases, which are endemic in 98 countries, reaching up to 1.2 million new cases per year, affecting mainly poor and marginalized populations, with 90% of cases ocurring in just six countries: India, Bangladesh, Sudan, South Sudan, Brazil and Ethiopia [1]. The clinical manifestations of the complex of diseases can involve the skin, with local (cutaneous), diffuse (diffuse cutaneous) or disfiguring lesions (mucocutaneous), or the viscera (liver, spleen and bone marrow), leading to death if untreated. It is caused by parasitic protozoa belonging to the genus *Leishmania*, transmitted to humans via the bites of sandflies.

In the decade of the 1940s, pentavalent antimony (Sb(V)) complexes, including meglumine antimoniate (MA) and sodium stibogluconate, were introduced as therapeutics for leishmaniasis. Even though pentavalent antimonials are still the first-line drugs against all forms of leishmaniasis in several countries, their use in the clinical setting shows several limitations [2]. These compounds have to be given parenterally, daily, for at least three weeks (typically, 20 mg of Sb/kg/day for 20–30 days). Antimony therapy is often accompanied by local pain during intramuscular injections and by systemic side effects, requiring very careful medical supervision. Even if the antimonial drugs remain an essential part of the treatment of visceral leishmaniasis in South America and Africa, by the end of the twentieth century, their efficacy in Bihar, which houses 90% of India’s visceral leishmaniasis cases, had decreased to cure rates of less than 50%, due to the widespread resistance to pentavalent antimonials. Consequently, use of antimonial drugs is no longer recommended in the Indian subcontinent [3]. At present, miltefosine (hexadecylphosphocholine) is the only drug available for oral treatment of leishmaniasis. However, its teratogenicity restricts its use in women of childbearing age [3]. In addition, its long half-life (~150 h) and the 28-days treatment duration make the drug critically prone to low compliance and emergence of drug resistance [4]. In this context, new drugs must be developed that are capable of being administered orally with minimal medical supervision, at an affordable price.

Different strategies have been investigated by our group to enhance the oral bioavailability of pentavalent antimonials. One consisted in the formation of Sb(V) complexes with amphiphilic ligands [5]. Another strategy applied to MA refers to its depolymerization [6], as this compound consists of a mixture of oligomeric structures with the general formula (NMG–Sb)_n_ and (NMG–Sb)_n_–NMG where NMG represents N-methyl-D-glucamine [7]. Depolymerization was achieved through heating of MA aqueous solution followed by freeze-drying [6]. An even more effective process was the heating of MA in the presence of β-cyclodextrin (β-CD) [6,8]. The improved oral bioavailability of Sb from MA/β-CD compositions was attributed to the formation of a NMG-Sb-β-CD complex that maintains the antimonial compound depolymerized and sustainedly releases the low molecular-weight 1:1 Sb-NMG species [6,9]. However, the low solubility of β-CD was found to be a limitation for application in large animals [10]. This limitation was circumvented in part by increasing the Sb/β-CD molar ratio from 1:1 to 7:1 [10]. The proposed mode of action of MA/β-CD compositions suggested that other simpler sugars may also be able to improve the bioavailability of MA through complexation [11]. In the present work, ribose and maltose were investigated for their ability to form mixed complex with MA, sustainedly release MA and modulate the serum level of Sb after oral administration in mice. The results indicate that MA/maltose and MA/ribose compositions indeed act as sustained drug release systems, prolonging the serum level of Sb after oral administration in mice.

## 2. Results and Discussion

### 2.1. Characterization of the Interaction between MA and Sugars by Circular Dichroism

**Figure 1:**
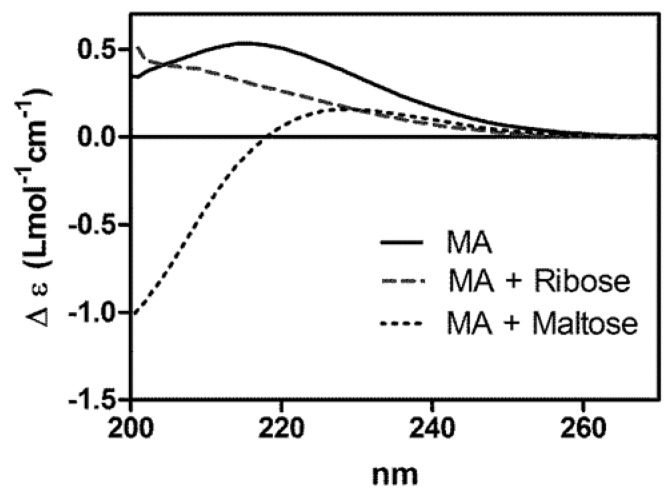
Circular dichroism spectra of MA and its association compounds with ribose and maltose. MA at 700 mmol/L of Sb in water was incubated with an equimolar amount of ribose or maltose for 3 h at 60 °C. Circular dichroism spectra were registered immediately after dilution at 5 mmol/L of Sb using 0.2 cm cuvette.

Evidence for the interaction of MA with ribose or maltose was first obtained by circular dichroism. Figure 1 shows the circular dichroism spectra of MA in the absence or presence of ribose or maltose. It is noteworthy that neither antimoniate alone, nor the sugars alone (NMG, ribose and maltose) contributed circular dichroism signals in this wavelength region (data not shown). The positive circular dichroism band of MA centered at 215 nm, characteristic of Sb-NMG complexes [6], suffered a decrease of intensity and a blue shift, upon mixing of MA with ribose. Furthermore, the presence of maltose resulted in a decrease of intensity of the positive band and the concomitant appearance of a negative band. This data clearly indicates a change in the environment of Sb(V) in the presence of the sugars, evidencing the occurrence of interactions between MA and the sugars.

This property of MA is consistent with our previous reports that Sb(V) can form complexes with β-CD and ribonucleosides [6,8,9,10,12]. Indeed, Sb(V) was found to bind to the glucose moiety of β-CD, as those encountered in maltose. In addition, NMR analyses suggested that Sb(V) binds to ribonucleosides through ribose –OH groups and via ring chelation at C2′ and C3′ [13,14].

### 2.2. Characterization of the Different Molecular Species in MA/Sugar Compositions

It is noteworthy that inorganic pentavalent antimonials, such as MA, usually consist in a mixture of several complexes of different order [7] and, consequently, can only be obtained in an amorphous state. In this context, mass spectrometry, HPLC and NMR are so far the only techniques that have been used successfully in the physicochemical characterization of these compounds [15]. In the present work, high performance liquid chromatography coupled to mass spectrometry (LCMS-IT-TOF) and UV detection was used to identify the main species present in the different MA/sugar compositions.

**Figure 2:**
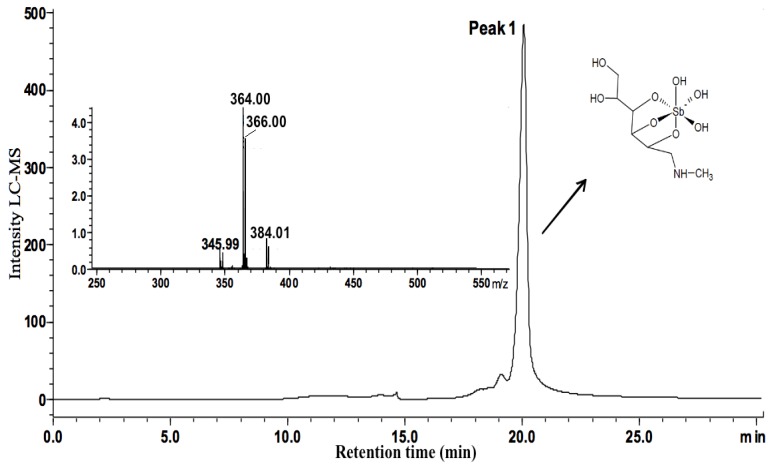
LCMS-IT-TOF spectra of the MA complex. Peak 1 shows the main event at 19.23–20.59 min retention time with ESI(−)MS peak at *m/z* 364 attributed to 1:1 Sb-NMG complex.

**Figure 3:**
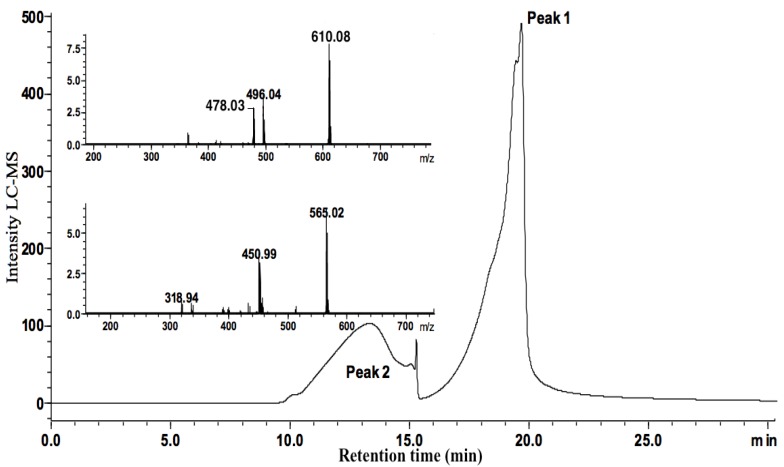
LCMS-IT-TOF spectra of the MA/ribose composition. Two main events showing retention times: at 18.08–19.52 min with ESI-MS peaks at *m/z* 478 and 610 characteristic of MA-ribose mixed complexes; at 12.38–14.02 min with ESI-MS peaks at *m/z* 450 and 565 corresponding to Sb/ribose complexes.

**Figure 4:**
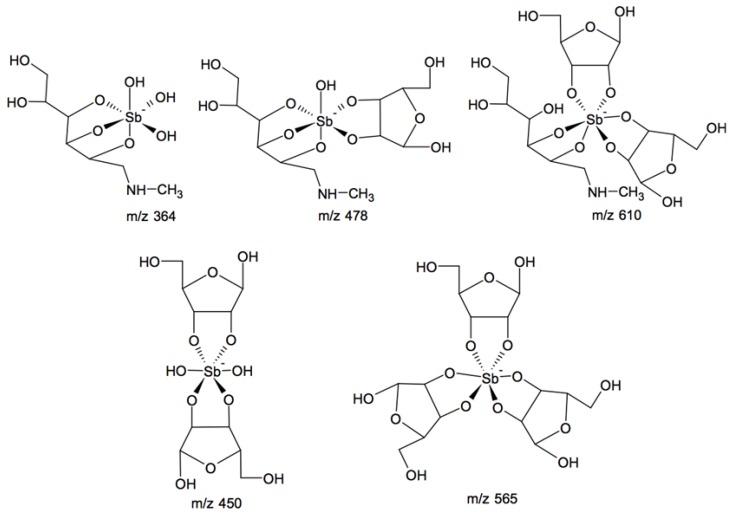
Structures proposed for the MA and MA-ribose mixed complexes on the basis of HPLC-IT-TOF experiments.

Figure 2 and Figure 3 show the chromatograms of MA and MA/ribose composition, respectively. MA displayed a main chromatographic event with 19.23–20.59 min retention time and an ESI-MS peak at *m/z* 364 characteristic of 1:1 Sb-NMG complex (structure shown in Figure 4) [7]. Furthermore, MA/ribose composition showed two main events with different retention times: the first event at 12.38–14.02 min with ESI-MS peaks at *m/z* 450 and 565 corresponding to Sb-ribose complexes; and the second event at 18.08–19.52 min with ESI-MS peaks at *m/z* 478 and 610 characteristic of MA-ribose mixed complexes. In this case, mixed complexes mean ternary ribose–Sb–meglumine complexes with covalent bonds between the different molecular entities. According to previous studies of the interaction of Sb(V) with ribonucleosides and ribose [12,13,16], these complexes most probably arise from the binding of Sb(V) to vicinal hydroxyl groups in the ribose molecule (Figure 4).

Table 1 summarizes the retention times and corresponding species determined in free MA and MA/ribose composition. On the other hand, the presence of ternary complexes could not be detected by ESI-MS in the chromatogram of MA/maltose composition. Indeed, peaks showing *m/z* 364 species were observed with different retention times (11.32–12.10; 18.71–19.00; 20.08–21.14 min).

**Table 1:** Ionic species identified by LCMS-IT-TOF in MA, MA/ribose and MA/maltose compositions.

Anionic Species	*m/z*	Retention Time (min)
**MA**		
[(NMG − 3H)Sb(OH)_3_ + H_2_O]^−^	382	16.17–16.51
[(NMG − 3H)Sb(OH)_3_ − H_2_O)]^−^	346	17.22–18.59
[(NMG − 3H)Sb(OH)_3_]^−^	364	19.23–20.59
**MA/ribose**		
[(2Ribose − 4H)Sb(OH)_2_]^−^	450	12.38–14.02
[(3Ribose − 6H)Sb(OH)_3_]^−^	565	12.38–14.02
[(NMG − 2H)Sb(2Ribose-4H)]^−^	610	18.08–19.52
[(NMG − 4H)Sb(O_3_H) + (ribose-2OH)]^−^	478	18.08–19.52
478 + H_2_O	496	18.08–19.52
**MA/maltose**		
[(NMG − 3H)Sb(OH)_3_]^−^	364	11.32–12.10
		18.71–19.00
		20.08–21.14

The presence of MA species that displayed retention times smaller than that of free MA (data not shown), suggests the existence of several unstable association compounds between maltose and MA, like for instance that reported previously in the MA/β-CD composition [8]. In the case of maltose, the lack of *cis* vicinal hydroxyl groups probably prevented the formation of covalent bonds with Sb(V). Thus, association compounds may have been formed through hydrogen bonding with MA.

### 2.3. Kinetics of Change of Sb Complexation State upon Dissolution of the MA/Sugar Compositions

**Figure 5:**
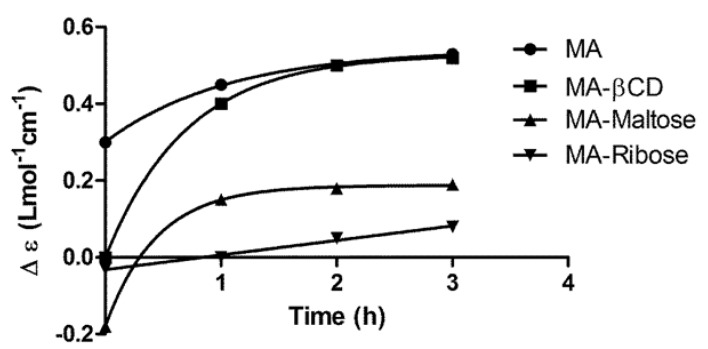
Kinetics of dissociation of MA-ribose, MA-maltose and MA-βCD mixed complexes at 37 °C after dilution in water at 5 mM Sb, followed by the circular dichroism signal at 215 nm.

In order to infer the change of Sb complexation state upon dilution of the MA/sugar compositions in biological fluids, the variation of the circular dichroism signal was evaluated as a function of time after dissolution of the freeze-dried composition in water and incubation at 37 °C. In this experiment, depolymerized MA and the MA/β-CD composition (prepared at a Sb/β-CD molar ratio of 7:1 [10]) were used as controls. As shown in Figure 5, the recovery of MA signal after 2 h incubation of MA/β-CD indicates that the association compound slowly releases MA after dissolution. Both the MA/ribose and MA/maltose compositions showed a slow increase in the circular dichroism signal. The stabilization of the circular dichroism signal of MA/maltose composition after 1 h incubation, at a value lower than that of MA, suggests a partial dissociation of the association compound(s). On the other hand, the MA/ribose composition showed no stabilization of the circular dichroism signal on the 3 h evaluation period, indicating a very slow change of Sb complexation state and the lack of recovery of the MA. Although this circular dichroism data suggested a partial and slow dissociation of the new compounds formed in the mixture, one could not infer about the dissociation of a specific complex, because the compositions consisted in mixtures of different compounds.

### 2.4. Serum Levels of Sb after Oral Administration in Mice

**Figure 6:**
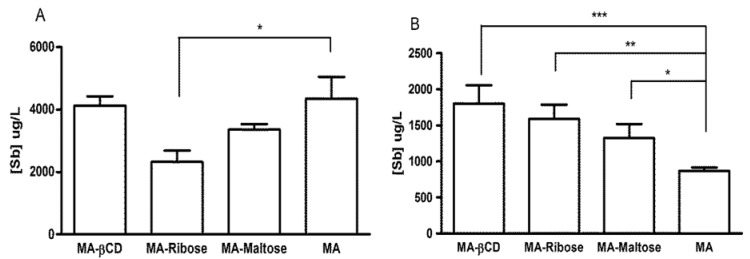
Serum concentrations of Sb in Swiss mice, 1 h (**A**) and 3 h (**B**) after oral administration of MA at 300 mg Sb/kg of body weight and different mixed antimony complexes with ribose, maltose or β-CD. * *p* < 0.05, ** *p* < 0.01, *** *p* < 0.001 for comparison between MA and the other groups, One-way ANOVA followed by Bonferroni post-test.

To evaluate the ability of the antimony(V) compositions to modulate the serum level of Sb after administration by the oral route, MA/ribose and MA/maltose compositions were given orally to Swiss mice at 300 mg Sb/kg of body weight and Sb levels were determined in the serum after 1 and 3 h of administration. The times of 1 and 3 h were chosen as indicators of the serum peak and elimination phase, respectively [10]. MA in the depolymerized form and the 7:1 MA/β-CD composition were also used as reference compositions capable of promoting elevated serum levels of Sb, compared to conventional MA [6,10]. Figure 6A shows that after 1 h of administration, the MA/maltose composition exhibited similar serum Sb level as depolymerized MA and MA/β-CD. On the other hand, MA/ribose composition promoted a significantly lower serum level of Sb, when compared to depolymerized MA (*p* < 0.05, One-way ANOVA).

After 3 h of administration, both MA/ribose and MA/maltose compositions promoted significantly greater serum levels of Sb compared to depolymerized MA (*p* < 0.05, One-way ANOVA) and similar level as MA/β-CD (Figure 6B).

This data indicates that the MA/maltose and MA/ribose compositions significantly modulate the oral bioavailability of Sb from MA, resulting in more sustained serum levels.

**Figure 7:**
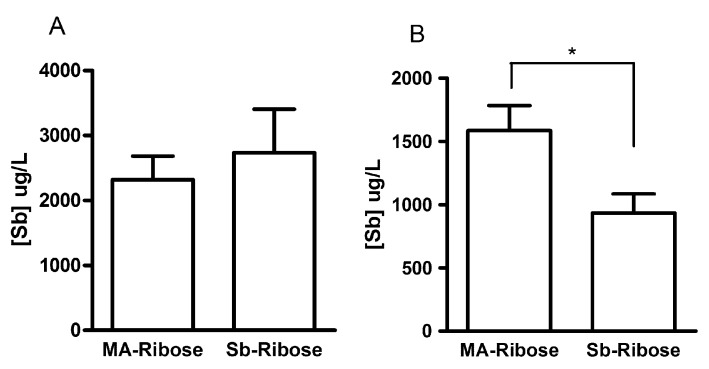
Serum concentrations of Sb in Swiss mice, 1 h (**A**) and 3 h (**B**) after oral administration of MA/ribose composition and Sb-ribose complex at 300 mg Sb/kg of body weight. * *p* < 0.05, Test t.

To determine whether direct complexation of Sb(V) to ribose would produce the same pharmacokinetic profile of Sb as that observed after the MA/ribose composition, Sb concentrations were determined 1-h and 3-h after administration of the Sb-ribose complex by oral route. The results presented in Figure 7 indicates that significantly lower level of Sb was achieved after 3 h from the Sb-ribose complex, when compared to MA/ribose composition (*p* < 0.05, student t test).

Thus, in contrast to MA/ribose and MA/maltose compositions, the Sb-ribose complex was unable to promote sustained levels of Sb in the serum. One can reasonably assume that mixed complexes have to dissociate and release the low molecular-weight Sb-NMG complex to allow for the gastrointestinal absorption of Sb. Thus, the low Sb concentration found 1 h after oral MA-ribose and the high Sb concentration after 3 h are consistent with the slow dissociation rate of this mixed complex.

## 3. Experimental

### 3.1. Materials

Antimony pentachloride (SbCl_5_), *N*-methyl-D-glucamine (NMG), β-cyclodextrin (β-CD), ribose and maltose were obtained from Sigma-Aldrich (St. Louis, MO, USA). All reagents were used without further purification.

### 3.2. Animals and Ethical Issues

Swiss mice female were obtained from CEBIO (Centro de Bioterismo, Instituto de Ciências Biológicas, Universidade Federal de Minas Gerais, Belo Horizonte, Brasil). Standard diet and tap water was supplied *ad libitum*. Experimental protocols were performed in accordance with the guidelines for the humane use of laboratory animals and received approval from the Ethics Committee in Animal Experimentation of the Federal University of Minas Gerais (protocol n° 004/03 and 142/2006).

### 3.3. Preparation of MA and MA/Sugar Complexes

MA was synthesized as previously described [17,18], from a mixture of NMG and freshly precipitated, hydrated Sb pentoxide, which was obtained from SbCl_5_ hydrolyzed in water. The resulting product contained 29% of Sb by weight, as determined by inductively coupled plasma optical emission spectrometry, and consisted of an equimolar mixture of Sb and NMG. The Sb-ribose complex was synthesized using the same procedure as for MA. The resulting white amorphous solid was found to contain one water molecule of hydration. Anal. Found: C, 16.24; H, 3.28; Sb, 31.93. Calc. for [C_5_H_12_O_5_Sb·H_2_O]K (1:1 Sb-ribose complex): C, 15.92; H, 3.18; Sb, 32.30%.

The MA/sugar compositions were prepared by dissolving the sugar powder in a solution of MA in water at 0.7 mol/L of Sb, at a final Sb/sugar molar ratio of 1:1. The MA/sugar mixture was then heated at 60 °C for 3 h under stirring, maintaining the pH around 7.0 by addition of HCl or KOH. The resulting solutions were frozen in liquid nitrogen and dried overnight using Liotop freeze-dryer (Sao Carlos, SP, Brazil). Depolymerized MA and MA/β-CD composition (Sb/β-CD molar ratio of 7:1) were prepared using the same protocol [6,10]. The amount of Sb in the different MA compositions was determined by electrothermal atomic absorption spectrometry (ETAAS) using a Perkin-Elmer AA600 spectrometer (Shelton, CT, USA).

### 3.4. Circular Dichroism Characterization

Circular dichroism spectra were recorded on a Chirascan spectropolarimeter (Applied Photophysics, Surrey, UK). This study explored the characteristic circular dichroism signal of MA, as described previously [6]. Evidence for the interaction of MA with each sugar was obtained by recording the circular dichroism spectrum of corresponding MA/sugar composition in water and comparing it to that of depolymerized MA.

The kinetic of dissociation of the MA/sugar composition was investigated by recording circular dichroism spectra at different time intervals after dissolution of the MA-sugar powder at the final concentration of 5 mmol/L of Sb in water and incubation at 37 °C.

The solutions were transferred to a 0.2 cm quartz cuvette and the circular dichroism spectrum was recorded in the 200–270 nm wavelength range. The circular dichroism signal is given as Δε, which is the differential molar dichroic absorption coefficient (Δε = ε_L_ − ε_R_ in Lcm^−1^ mol^−1^) and is expressed in terms of the molar concentration of Sb.

### 3.5. LCMS-IT-TOF Characterization of the Different Molecular Species

The MA/sugar compositions and depolymerized MA were analyzed by high-performance liquid chromatography coupled to mass spectrometry (LCMS-IT-TOF). Chromatographic conditions used for separation were: Shodex OHpak SB-804 HQ column (300 mm × 8.0 mm), UV–Vis detector at 220 nm, MilliQ water as mobile phase, room temperature, 0.2 mL/min flow-rate and 20 µL injection volume. Samples were solubilized at 1% (w/v) in MilliQ water. ESI–MS analysis used capillary cone heated at 200 °C, 1.63 kV spray voltage with nitrogen, interface voltage at −3.5 KV. The mass spectra were acquired in a range from *m/z* 50–1000 in the negative mode.

### 3.6. Serum Antimony Levels after Oral Absorption in Mice

Groups of Swiss mice (female, 25 ± 3 g) received by gavage the different MA compositions at 300 mg Sb/kg of body weight. Animals were sacrificed 1 or 3 h after administration by cervical dislocation after ketamine-xylazine anesthesia. Blood samples were collected by cardiac puncture and the serum was recovered and frozen at −20 °C.

Antimony was determined in diluted serum by electrothermal atomic absorption spectrometry (ETAAS) using a Perkin-Elmer AA600 graphite furnace atomic absorption spectrometer, as described previously [10]. The analytical method for determination of Sb in the serum was validated and showed suitable levels of precision (coefficient of variation [CV] 5%), accuracy (80% to 120% analyte recovery) and linearity (range of 10 to 180 g of Sb/L). The quantification limits of the analytical methods were 240 μg of Sb/L. Statistical analyses of serum Sb levels for comparison of the different compositions used One-Way ANOVA.

## 4. Conclusions

With the aim of increasing the serum level of Sb after administration of MA by the oral route, as previously reported for the MA/β-CD composition, we have investigated here the formation of complexes between MA and maltose or ribose. LCMS-IT-TOF analyses of MA and their compositions with ribose and maltose demonstrated the formation of mixed or association complexes. Interestingly, these compositions sustainedly release MA upon dilution in water at 37 °C. After oral administration in mice, the compositions were found to promote more sustained serum level of Sb, when compared to depolymerized MA. The mixed or association complexes between MA and simple sugars, such as ribose and maltose, emerge as promising compositions for the oral treatment of leishmaniasis.
